# Longitudinal SARS‐CoV‐2 Antibody Response in Healthcare Workers: Benefit of Prior Infection and Heterologous Boosting on Anti‐Spike IgG Immunity

**DOI:** 10.1111/irv.70202

**Published:** 2026-01-05

**Authors:** Els Van Nedervelde, Ellen Vancutsem, Deborah De Geyter, Diederik De Cock, Rhea Buttiens, Thessa Laeremans, Joeri L. Aerts, Sabine D. Allard

**Affiliations:** ^1^ Department of Internal Medicine and Infectiology Vitality Research Group (MIPI), Vrije Universiteit Brussel, Universitair Ziekenhuis Brussel Brussels Belgium; ^2^ Neuro‐Aging and Viro‐Immunotherapy Research Group (NAVI), Vrije Universiteit Brussel Brussels Belgium; ^3^ Clinical Biology, Laboratory of Microbiology and Infection Control Vrije Universiteit Brussel, Universitair Ziekenhuis Brussel Brussels Belgium; ^4^ Biostatistics and Medical Informatics Research Group, Department of Public Health, Faculty of Medicine and Pharmacy Vrije Universiteit Brussel Brussels Belgium

**Keywords:** adenovirus, antibodies, COVID‐19 vaccines, healthcare workers, mRNA, SARS‐CoV‐2, serological tests, vaccination, vaccines, viral

## Abstract

**Background and Objectives:**

Different COVID‐19 vaccine platforms elicit variable immune responses, influenced by prior infection and booster vaccination. We aimed to compare the humoral immune responses elicited by mRNA and adenoviral vector (Ad‐vector) COVID‐19 vaccines in hospital employees and to assess the possible impact of prior SARS‐CoV‐2 infection on these responses.

**Methods:**

We performed a prospective observational cohort study by recruiting employees of the Universitair Ziekenhuis Brussel who were vaccinated with an mRNA or Ad‐vector vaccine. We assessed anti‐spike (S) IgG and neutralising capacity at 1, 6 and 12 months (post‐mRNA booster). Anti‐S and anti‐NCP IgG were measured by chemiluminescent microparticle immunoassay, and neutralising capacity was assessed using Genscript's cPASS. Non‐parametric group comparisons used the Mann–Whitney U test, complemented by multiple linear regression.

**Results:**

Following RVR, mRNA‐vaccinated individuals (*n* = 380) exhibited higher anti‐S IgG titres and neutralising capacity compared to those who received Ad‐vector vaccines (*n* = 200). After the booster, anti‐spike IgG remained higher in mRNA‐vaccinated individuals than in Ad‐vector recipients (*q* < 0.001); Ad‐vector vaccinated individuals showed superior neutralising capacity. Natural SARS‐CoV‐2 infection prior to vaccination had varying impact on anti‐S IgG and neutralising capacity, depending on vaccination type and time points.

**Conclusion:**

Our findings underscore that both vaccine platforms and prior infections shape the magnitude and quality of the humoral immune response, highlighting the importance of considering priming strategy, booster design and hybrid immunity when optimising COVID‐19 vaccination schedules for durable protection.

## 1 Introduction

Since the emergence of SARS‐CoV‐2 in early 2020, the COVID‐19 pandemic has caused over 640 million confirmed cases and 6.6 million deaths worldwide [1]. The virus spreads through droplets, close contact and aerosols, placing intense pressure on healthcare systems. Understanding immune protection, both from infection and vaccination, has been critical to reducing transmission and protecting vulnerable populations [2].

The development of COVID‐19 vaccines significantly altered the trajectory of the pandemic. Among the most widely used platforms, mRNA and adenoviral vector (Ad‐vector) vaccines differ in immunological profiles and mechanisms of action [3, 4, 5]. mRNA vaccines typically elicit higher anti‐spike IgG titres, though evidence on long‐term durability is conflicting. In contrast, Ad‐vector vaccines induce stronger T‐cell responses and broader memory formation [6, 7, 8, 9]. However, comparative long‐term data on humoral immunity, particularly beyond the primary series and in high‐risk populations like healthcare workers (HCWs), remain scarce. Moreover, the impact of prior infection on the magnitude and persistence of vaccine responses appears to confer more sustained antibody responses, yet few longitudinal studies have tracked its kinetics across vaccine types [10, 11, 12, 13, 14].

Antibody levels generally peak within weeks post‐vaccination and decline variably in decay rates depending on vaccine platform, age, sex and prior infection [15, 16]. Despite high short‐term seroconversion rates, the extent of antibody waning, particularly beyond 6 months, remains unclear, underscoring the need for comprehensive, real‐world longitudinal data.

HCWs are particularly relevant for immunological surveillance due to their increased risk of SARS‐COV‐2 exposure. Despite personal protective equipment use, HCWs have experienced significant infection rates, especially early in the pandemic [2]. In a previous cross‐sectional study at Universitair Ziekenhuis Brussel (UZ Brussel), a university hospital in the Belgian capital of Brussels, we observed a 7.4% seroprevalence among hospital employees during the first wave (May–June 2020) [17].

In the present longitudinal study, we investigated the humoral immune response among UZ Brussel HCWs vaccinated with either an mRNA or Ad‐vector COVID‐19 vaccine, with or without prior SARS‐CoV‐2 infection. By analysing antibody titres and neutralising capacity at multiple time points post‐vaccination and post‐booster, we aimed to characterise the durability and quality of immune responses and assess the impact of hybrid immunity in a real‐world clinical setting.

## 2 Materials and Methods

### 2.1 Study Design

**FIGURE 1:**
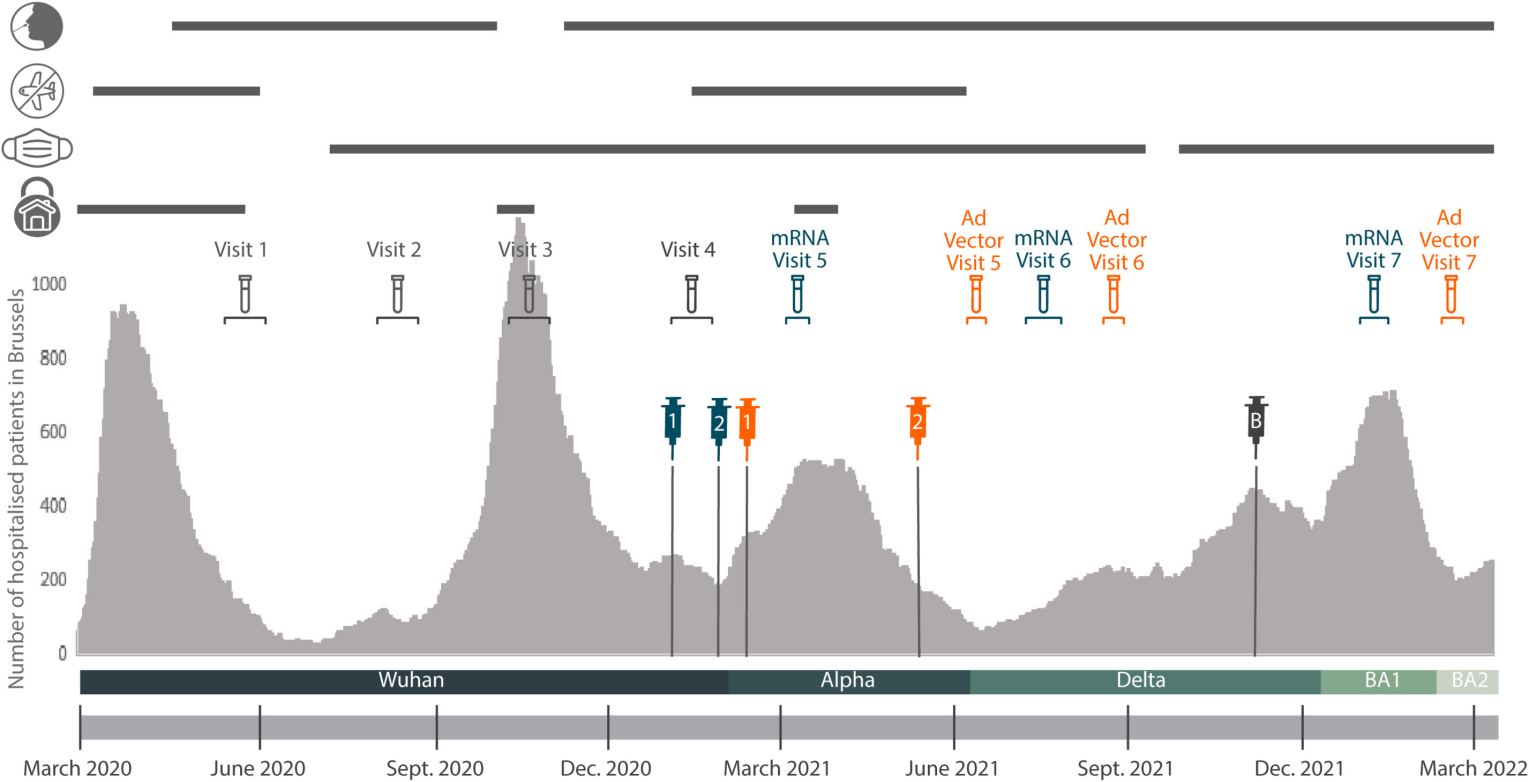
Vaccination cohorts superimposed on pandemic evolution. Number of COVID‐19‐positive hospital admissions in Brussels (March 2020–April 2022), regardless of primary diagnosis. The figure shows government‐mandated protective measures, including testing, travel bans, mask mandates and lockdowns [18]. Baseline blood sampling (‘Visit 4’) occurred before vaccination, while ‘Visit 5’ followed full mRNA/Ad‐vector vaccination. ‘Visit 6’ and ‘Visit 7’ represent samples taken 6 and 12 months post‐vaccination. Syringe icons indicate vaccinations: blue (mRNA), orange (Ad‐vector) and grey (mRNA boosters). The horizontal bar below marks dominant variant of concern (VOC) waves in Belgium [19].

This monocentric interventional prospective cohort study included participants between 19 May and 12 June 2020, as previously described [17]. UZ Brussel employees were eligible after signing an informed consent form. Blood sampling was done at inclusion (Visit 1) and at 2, 6 and 9 months (Visits 2–4) (Figure 1). Demographic data were collected at baseline. Participants could join subsequent visits, even if they missed earlier ones. In January 2021, SARS‐CoV‐2 vaccination became available. UZ Brussel launched a staff vaccination campaign, prioritising HCWs in direct patient care, per government guidelines. Consequently, at Visit 4, a subset of participants was invited for a follow‐up. Initially, only Comirnaty (Pfizer/BioNTech) was available as a vaccine. About 6 weeks later, Vaxzevria (AstraZeneca/Oxford University) became available, accelerating the vaccination campaign. Consequently, Visit 4 occurred just before vaccination for mRNA recipients and 6 weeks prior for Ad‐vector recipients. Second doses followed at 4 (mRNA) and 12 (Ad‐vector) weeks. Over the following 12 months, participants were invited for three additional blood draws (Visits 5–7) (Figure 1). Between Visits 6 and 7, employees were offered a booster vaccine with Comirnaty or Spikevax (Moderna); the latter was provided by UZ Brussel.

Figure 1 also illustrates the epidemiological evolution of COVID‐19 and related public health measures in Belgium during the study period.

The COVEMUZ study was approved by the UZB Brussel ethics committee (BUN No. 1432021000432 and 1432021000488).

### 2.2 SARS‐CoV‐2‐Specific Antibody Analysis

Anti‐nucleocapsid (NCP) measurements were used to distinguish between COVID‐19 naive and previously exposed employees in the pre‐vaccination cohort and to monitor new infections. Anti‐NCP IgG was determined using the SARS‐CoV‐2 IgG CMIA on Alinity i (Abbott Diagnostics, Illinois, USA) with a positive cut‐off (S/Co) ≥ 1.4, according to the manufacturer's instructions. Once identified as COVID‐19 previously exposed, all subsequent samples were screened for anti‐S IgG using the SARS‐CoV‐2 IgG II Quant test (Abbott, Illinois, USA), cut‐off ≥ 50 AU/mL, based on the manufacturer's instructions. Anti‐S IgG positive samples were further analysed for neutralising capacity using the cPASS ELISA (Genscript, New Jersey, USA), which quantifies SARS‐CoV‐2‐specific antibodies blocking the interaction between the RBD–ACE2 interaction (positive if inhibition ≥ 30%). All post‐vaccination samples were assessed for anti‐NCP, anti‐S IgG and neutralising antibodies. In this study, anti‐S IgG and neutralising antibody assays evaluated the vaccination‐induced responses, while anti‐NCP served as a marker of natural infection.

### 2.3 Statistical Analysis

Non‐parametric analyses were conducted in GraphPad Prism version 9 for Windows (GraphPad Software, Boston, USA). As data were not normally distributed, group comparisons were performed using the Mann–Whitney U test or Kruskal–Wallis test. Multiple comparisons were adjusted using the false discovery rate (FDR), with statistical significance set at *q* < 0.05 [20].

Multiple linear regression was performed in IBM SPSS Statistics for Windows, Version 29.0.0.0 (IBM Corp., Armonk, NY, USA) to adjust for confounding. Missing values were addressed by multiple imputation (10 imputations), including all available variables. Regression models were fitted on pooled imputed datasets, with vaccine brand as the main predictor and age and sex as covariates.

## 3 Results

### 3.1 Demographics

At study initiation, 2662 of the 3800 UZ Brussel employees enrolled in the COVEMUZ cohort, previously described [17]. This study focused on comparing humoral responses in a subgroup of 580 participants from our large cohort: 380 received the mRNA recommended vaccination regimen (RVR), and 200 received the Ad‐vector RVR (Figure 1 and Table S1). Median age was 41 in both groups, with non‐significant differences by vaccine type or prior infection status (Figure S1). Women were overrepresented (> 4:1 or higher; Table 1). Most participants worked ≥ 0.8 FTE supporting reliable follow‐up.

**TABLE 1:** Demographics and serological data of study participants.

	mRNA vaccinated employees			Ad‐vector vaccinated employees			Total
	mRNA vaccinated employees total	COVID‐19 naive	COVID‐19 previously exposed	Ad‐vector vaccinated employees total	COVID‐19 naive	COVID‐19 previously exposed	
Number of employees (%)	**380**	289 (76.1)	91 (23.9)	**200**	164 (82)	36 (18)	**580**
Age, years (median; min–max)	**41 (22–64)**	42 (23–64)	40 (22–64)	**41 (23–56)**	41 (23–56)	42 (26–54)	**41 (22–64)**
Female (%)	**291 (76.6)**	219 (75.8)	72 (79.1)	**158 (79.0)**	126 (76.8)	32 (88.9)	**449 (77.4)**
Occupation							
Physician (%)	**54 (14.2)**	42 (14.5)	12 (13.2)	**3 (1.5)**	3 (1.8)	0 (0)	**57 (9.8)**
Nurse (%)	**281 (73.9)**	210 (72.7)	71 (78.0)	**14 (7.0)**	12 (7.3)	2 (5.6)	**295 (50.9)**
Pharmacist (assistant) (%)	**0 (0)**	0 (0)	0 (0)	**2 (1.0)**	2 (1.2)	0 (0)	**2 (0.3)**
Paramedical^a^ (%)	**28 (7.4)**	23 (8.0)	5 (5.5)	**33 (16.5)**	23 (17.7)	4 (11.1)	**61 (10.5)**
Non‐medical^b^ (%)	**17 (4.5)**	14 (4.8)	3 (3.3)	**148 (74.0)**	118 (72.0)	30 (83.3)	**165 (28.4)**
Percentage of employment							
0.8–1 FTE (%)	**247 (65.0)**	190 (64.7)	57 (6.6)	**135 (67.5)**	118 (72.0)	17 (47.2)	**382 (65.9)**
0.5–0.8 FTE (%)	**125 (32.9)**	92 (31.8)	33 (36.3)	**62 (31.0)**	45 (27.4)	17 (47.2)	**187 (32.2)**
< 0.5 FTE (%)	**5 (1.3)**	5 (1.7)	0 (0.0)	**3 (1.5)**	1 (0.6)	2 (5.6)	**8 (1.4)**
Booster vaccination							
mRNA (Comirnaty) (%)	**39 (10.3)**	29 (10.0)	10 (11.0)	**7 (3.5)**	5 (3.0)	2 (5.6)	**46 (7.9)**
mRNA (Spikevax) (%)	**258 (67.9)**	197 (68.2)	61 (67.0)	**142 (71.0)**	114 (69.5)	28 (77.8)	**400 (69.0)**
No booster (%)	**3 (0.79)**	3 (1.04)	0 (0.0)	**1 (0.5)**	0 (0.0)	1 (2.8)	**4 (0.7)**
LTFU (%)	**80 (21.1)**	60 (20.8)	20 (22.0)	**50 (25.0)**	45 (27.4)	5 (13.9)	**130 (22.4)**
Anti‐NCP positive							
Visit 4 (%)	**51 (13.4)**	0 (0.0)	51 (56.0)	**25 (12.5)**	0 (0.0)	25 (69.4)	**76 (13.1)**
Visit 5 (%)	**36 (9.5)**	0 (0.0)	36 (39.6)	**14 (8.3)**	4 (2.9)	10 (3.3)	**40 (9.1)**
Visit 6 (%)	**18 (5.9)**	3 (1.3)	15 (19.7)	**9 (5.6)**	5 (3.8)	4 (12.5)	**27 (5.8)**
Visit 7 (%)	**54 (18.0)**	40 (17.5)	14 (19.7)	**45 (30.0)**	34 (28.6)	11 (35.5)	**99 (22.0)**
Anti‐spike IgG titre (median, AU/mL)							
Visit 4 [IQR]	**206 [113–618]**	/	206 [113–618]	**306 [145–746]**	/	306 [145–746]	**229 [113–663]**
Visit 5 [IQR]	**11,839 [6550–19,668]**	9353 [5435–15,095]	24,485 [16,313–36,379]	**1469 [896–2787]**	1307 [791–2108]	4844 [2483–13,592]	**8984 [3738–16,853]**
Visit 6 [IQR]	**1453 [723–3291]**	1096 [620–1814]	5210 [3006–8635]	**756 [384–1739]**	606 [359–993]	3218 [1788–6670]	**1247 [627–2855]**
Visit 7 [IQR]	**24,611 [12,860–40,000]**	28,831 [13,918–40,000]	18,979 [10,326–28,912]	**10,690 [5928–22,822]**	9800 [5486–23,875]	13,522 [9496–20,299]	**20,299 [10,017–38,525]**

Abbreviations: Ad‐vector, adenoviral vector; FTE, full‐time equivalent; IQR, interquartile range; LTFU, lost to follow up (no sample collection after booster vaccination); RVR, recommended vaccination regimen.

^a^ Paramedical: physiotherapist, occupational therapist, psychologist, lab technician, research assistant.

^b^ Non‐medical: employees working in logistics, morgue, facility services, IT, management or maintenance.

### 3.2 Higher Anti‐S IgG in mRNA Vaccinated Employees Compared to Those Vaccinated With Ad‐Vector

**FIGURE 2:**
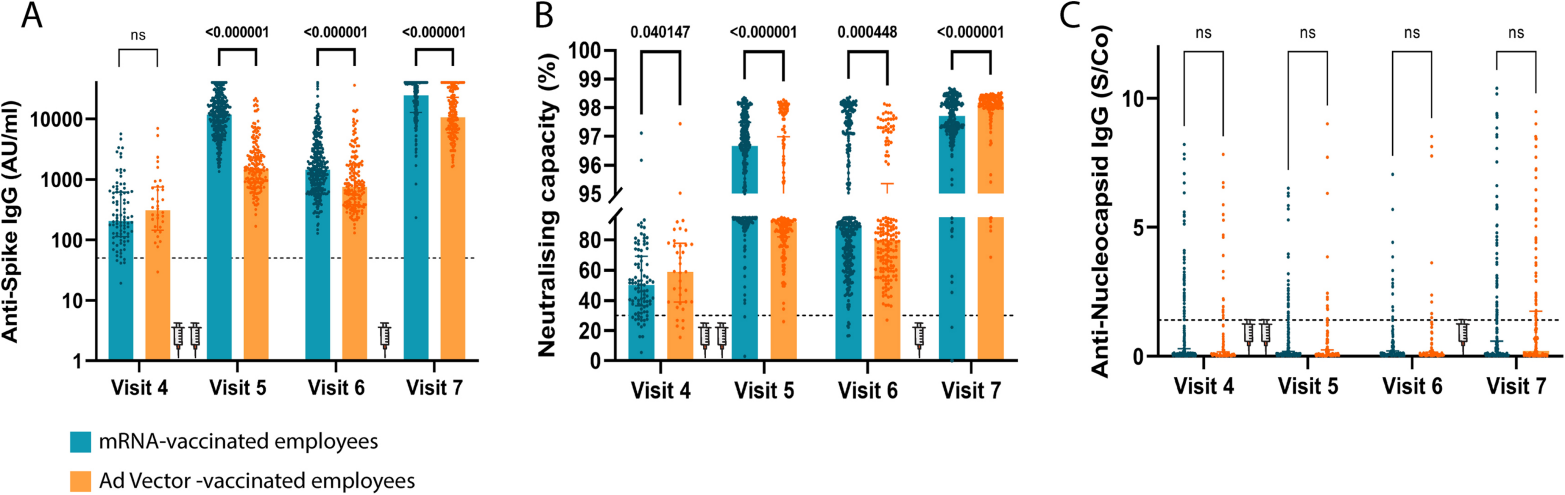
Comparison of humoral immune response within vaccination cohorts. Comparison of anti‐S IgG titres (A), anti‐S neutralising capacity (B) and anti‐NCP IgG levels (C) between employees vaccinated with mRNA (blue bars) and Ad‐vector (orange bars). Visit 4: at baseline (i.e., prior vaccination), Visit 5: 4–5 weeks after the second vaccine dose, Visit 6: 6 months after the first vaccine dose, and Visit 7: 12 months after the first vaccine dose. *q*‐values indicate the difference between the vaccination groups. Syringes in the graphs indicate vaccination. Booster vaccine (3rd syringe) was mRNA‐based in all participants. Bars indicate the median in each group. Mann–Whitney test was used, and statistical significance was accepted when *p* < 0.05. ns, not significant; S/Co, signal/cut‐off.

Pre‐vaccination anti‐NCP values showed 24% (mRNA‐vaccine recipients) and 18% (Ad‐vector vaccine recipients) previously exposed employees (Table S2). Baseline, anti‐S IgG titres were comparable (*q* = 0.16). Post‐vaccination, anti‐S IgG titres were significantly higher in the mRNA group (*q* < 0.001 and *q* = 0.02, Table 1, Figure 2, Table S3). Multiple linear regression analysis confirmed these patterns (*p* < 0.001 and *p* = 0.04, Table S5). Neutralising capacity was comparable pre‐vaccination (Figure 2B). Among previously exposed individuals, no significant difference was observed at Visit 5, but neutralising capacity was higher in the mRNA‐vaccinated group at Visit 6 (*q* = 0.012). However, adjusted linear regression analyses showed no significant post‐vaccination differences at either time point.

Despite stronger anti‐S IgG and neutralising capacity in the mRNA group, no statistically significant differences in subsequent SARS‐CoV‐2 infections were observed (Figure 2C), suggesting that higher antibody titres did not translate into better protection within the study timeframe.

### 3.3 Prior SARS‐CoV‐2 Infection Boosts Antibody Responses Post‐Vaccination

**FIGURE 3:**
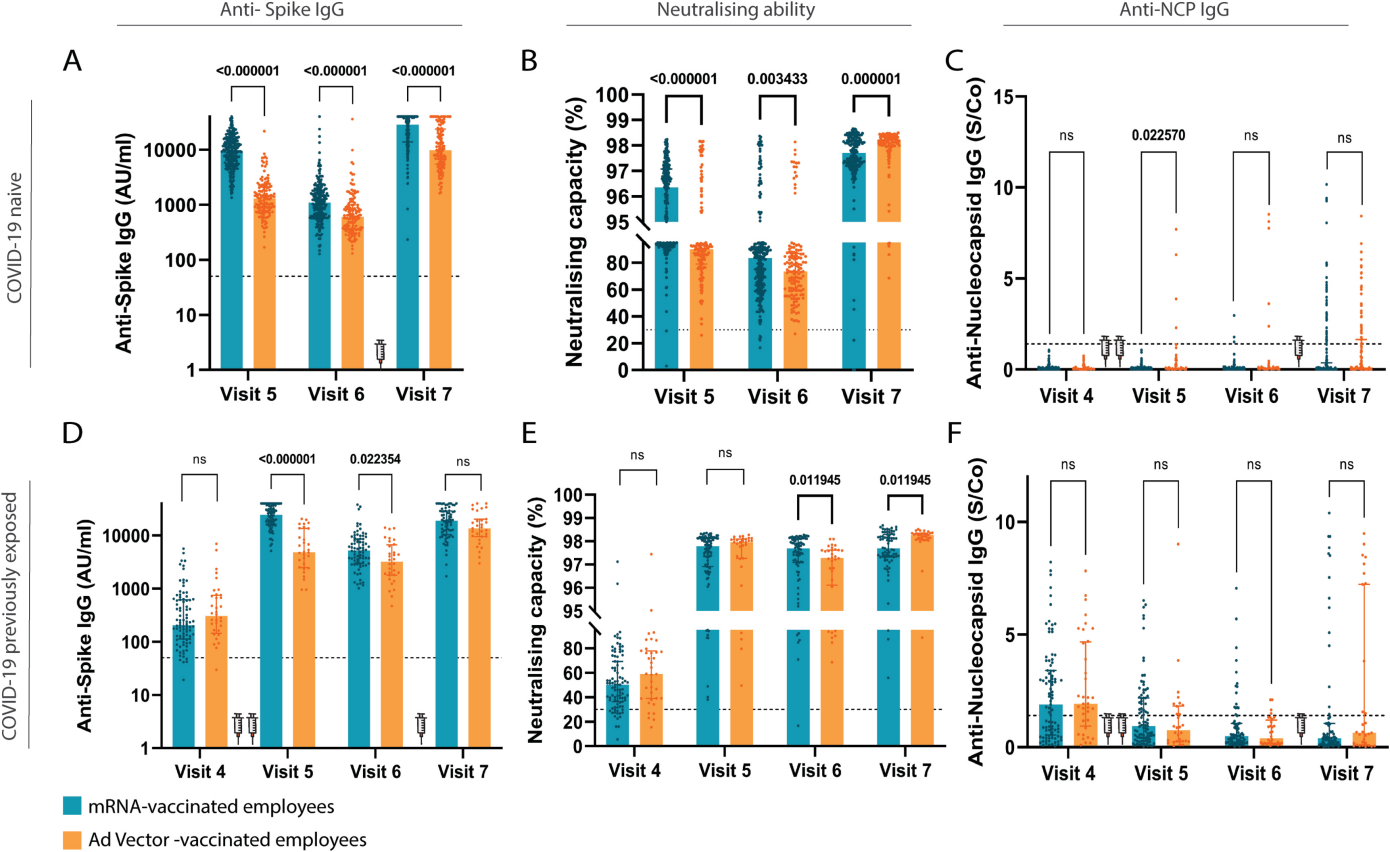
Comparison of humoral immune response between COVID‐19 naive and COVID‐19 previously exposed employees. COVID‐19 naive (A–C) and COVID‐19 previously exposed (D–F) participants are shown. (A) and (D) show anti‐S IgG titre, panels (B) and (E) show the neutralising capacity and panels (C) and (F) show the anti‐NCP IgG titres. Participants were tested before vaccination (i.e., at baseline, Visit 4) as well as 4–5 weeks (Visit 5), 6 (Visit 6) and 12 months (Visit 7) after full vaccination. The two first syringes indicate RVR with mRNA or Ad‐vector vaccine. The third syringe, between 6 and 12 months, indicates mRNA booster vaccination. Bars indicate the median in each group. Mann–Whitney test was used, and significance was accepted when *p* < 0.05. ns, not significant; S/Co, signal/cut‐off.

We evaluated the potential impact of prior SARS‐CoV‐2 infection on vaccine‐induced humoral responses. Participants were categorised as naive or previously exposed based on baseline (i.e., before vaccination) anti‐NCP antibodies (Figure 3). Naive mRNA recipients showed consistently higher anti‐S IgG and neutralising capacity than naive Ad‐vector recipients (Figure 3A,B). In line with the observation that mRNA vaccination generally elicited stronger humoral responses, no differences in anti‐NCP IgG were seen in COVID‐19–naive participants. However, among previously exposed individuals, median anti‐NCP IgG at Visit 5 was slightly higher in Ad‐vector recipients (*q* = 0.023) (Figure 3C), suggesting prior infection may modulate vaccine platform effects, though small sample size warrants caution.

Adjusted linear regression analysis confirms higher anti‐NCP IgG in Ad‐vector recipients at Visit 5 (*p* < 0.001, *β* = 0.21, Tables S5 and S7), supporting the interpretation that mRNA vaccination conferred stronger protection against SARS‐CoV‐2 infection in naive individuals.

At Visit 5, previously exposed mRNA recipients displayed higher anti‐S IgG titres than Ad‐vector recipients (*p* < 0.001; Figure 3D), a difference that persisted at Visit 6 (*p* = 0.41) but not at Visit 7 (*p* = 0.099). Regression analysis confirmed these findings (*β* = −16,026.4 and *β* = −2206.5), respectively. At 12 months (Visit 7), the regression model showed no significant difference, consistent with the Mann–Whitney U analysis. The use of both statistical approaches allowed us to detect distribution‐sensitive group differences while also adjusting for individual‐level covariates, thereby enhancing the robustness and interpretability of the results.

Neutralising capacity was similar at Visit 5, higher for mRNA recipients at Visit 6 (< 0.05) and higher for Ad‐vector recipients at Visit 7 (*q* < 0.05) (Figure 3E). However, the linear regression model found no significant differences in neutralising capacity at any of the time points, underscoring the added value of combining statistical approaches for robust interpretation. Anti‐NCP IgG levels remained comparable between vaccine groups among previously exposed participants (Figure 3F).

These findings highlight that prior infection enhanced vaccine‐induced responses, particularly after mRNA vaccination. In naive participants, mRNA vaccines elicited stronger humoral responses than Ad‐vector vaccines. Among previously exposed individuals, differences were less pronounced once confounders were considered. By 12 months, responses had largely converged, underscoring the waning of early differences and the importance of durability in vaccination strategies.

### 3.4 Influence of Basic Vaccination Regimen on Humoral Immune Response Following mRNA Booster

Six months after RVR, employees received an mRNA booster per governmental guidelines. Most received their booster (Spikevax) at the UZ Brussel, while others received Comirnaty or Spikevax at their local vaccination centre (Table 1).

**FIGURE 4:**
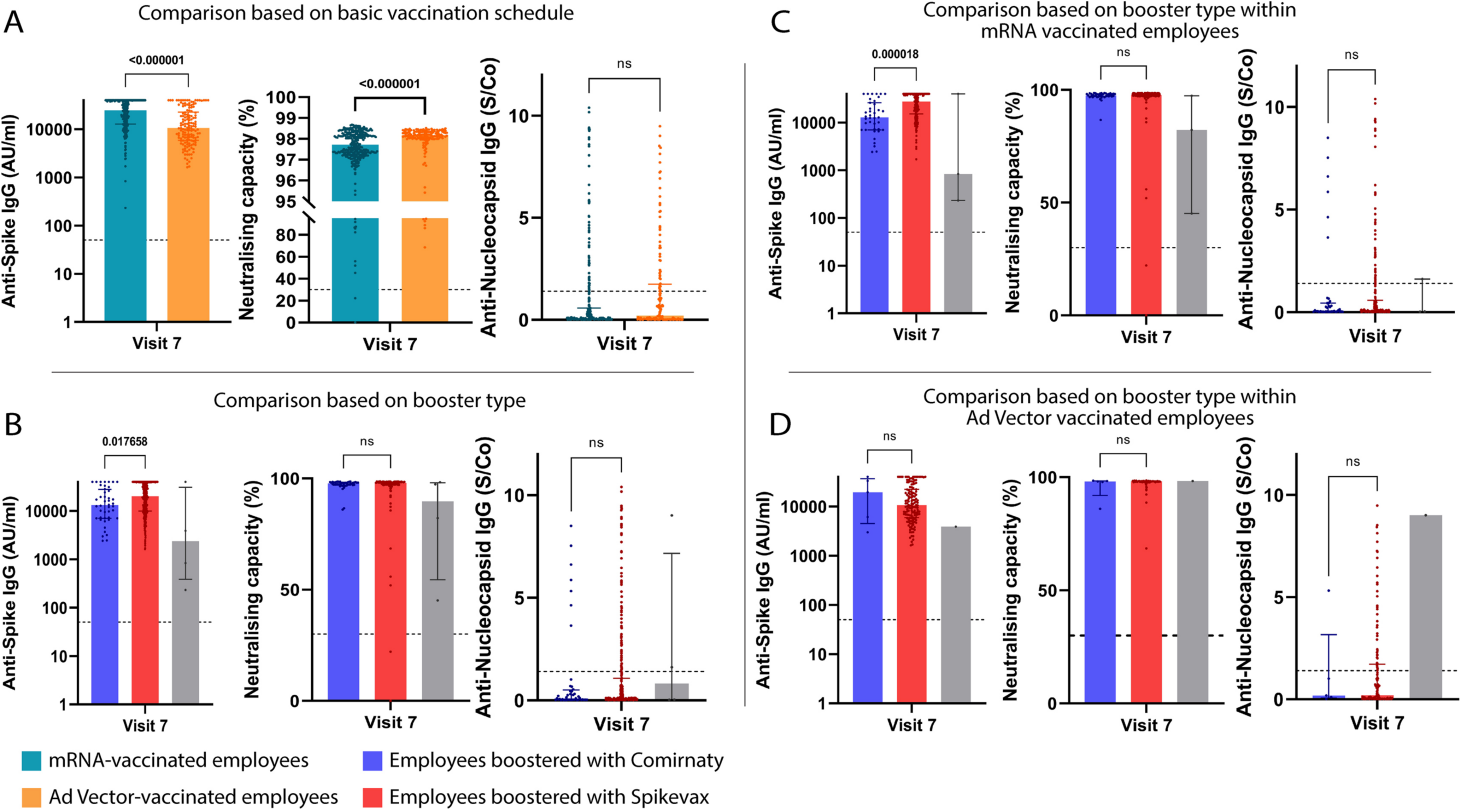
Antibody response in relation to the administered booster, with respect to the recommended vaccination regimen. Anti‐S titres, neutralising capacity and Anti‐NCP levels are shown. (A) shows the booster‐induced humoral responses for the two RVR, i.e., mRNA or Ad‐vector‐based vaccination. (B) shows humoral responses between participants receiving a Spikevax booster (red bars), a Comirnaty booster (blue bars) or no booster (grey bars) following basic vaccination. (C) and (D) show the differences in humoral immune responses post‐booster (Spikevax: red bars; Comirnaty: blue bars; no booster: grey bars) in relation to the RVR (panel C: mRNA vaccination, D: Ad‐vector‐vaccination). Bars indicate the median in each group. Mann–Whitney test was used, and significance was accepted when *p* < 0.05. ns, not significant; S/Co, signal/cut‐off.

Post‐mRNA booster, and irrespective of the type of booster received, mRNA‐RVR recipients displayed higher anti‐S IgG titres (*q* < 0.001; Figure 4A, Table S4). However, the regression model showed the opposite: lower anti‐S IgG titres in mRNA‐RVR (*p* < 0.001; *β* = −7153.4, Tables S6 and S8). Neutralising capacity was higher in Ad‐vector‐RVR recipients (*q* < 0.001) but not significant in linear regression. This discrepancy may reflect subtle differences in immune response quality not captured by total anti‐S IgG titres alone or may be influenced by residual confounding or assay sensitivity. Anti‐NCP IgG was comparable across vaccine groups (Figure 4A).

Spikevax booster yielded higher anti‐S IgG titres than Comirnaty (*q* = 0.018; Figure 4B). Linear regression analysis showed the reverse (*p* = 0.009; *β* = 5471.1). Neutralising capacity and anti‐NCP IgG titres showed no significant differences (Figure 4B).

In mRNA‐RVR recipients, Spikevax booster induced higher anti‐S IgG titres (*q* < 0.001; Figure 4C) than a Comirnaty booster, but linear regression again showed the reverse (*p* < 0.001; *β* = 9523.8).

Neutralising capacity and anti‐NCP IgG remained similar across booster types. In Ad‐vector‐RVR recipients, no differences in booster responses were observed with either analysis.

These findings indicate that the recommended vaccination regimen (RVR) influences the booster‐induced humoral response, but outcomes vary depending on the statistical method used. While the Mann–Whitney U test suggested that mRNA‐RVR led to higher anti‐S IgG levels post‐booster and that Ad‐vector‐RVR was associated with slightly greater neutralising capacity, the linear regression model instead indicated the opposite, with no significant differences in neutralising capacity. Similarly, while the Mann–Whitney U test highlighted a superiority of Spikevax over Comirnaty in inducing anti‐S IgG—particularly in mRNA‐primed individuals—the regression model consistently showed the reverse. This highlights the complexity of vaccine‐induced immunity and the importance of applying complementary analytical approaches in real‐world cohorts.

## 4 Discussion

In this prospective study, 580 UZ Brussel employees were followed up for 20 months covering both RVR and mRNA booster. Among previously exposed participants, neutralising capacity was similar 1 month post‐RVR, but mRNA recipients had significantly higher anti‐S IgG titres. After boosting, Ad‐vector‐recipients showed higher neutralising capacity; RVR‐mRNA‐recipients maintained anti‐S IgG titres. However, linear regression analysis sometimes contradicted the Mann–Whitney U results, particularly for anti‐spike IgG, underscoring the influence of statistical approach and covariates.

The Mann–Whitney U test, which makes no assumptions about data distribution, is well suited for skewed antibody titres, while linear regression allows adjustment for confounders, such as age and sex. Despite attempts at data transformations (e.g., log or square root), none of them sufficiently normalised the distribution, supporting the use of non‐parametric tests. Whereas non‐parametric analyses are sensitive to distributional differences and highlight group‐level contrasts, regression models account for individual‐level covariates and yield more conservative estimates, particularly in heterogeneous real‐world cohorts. Reporting both approaches therefore provides complementary perspectives on the data.

Despite presuming higher occupational risk for HCWs, anti‐NCP seroconversion did not reflect this, likely due to stringent measures implemented at the UZ Brussel during the COVID‐19 pandemic. The fact that some nursing roles involved tasks without direct patient contact, such as data nurses, might also have influenced this discrepancy. This assertion is supported by the lack of a significant difference in anti‐NCP levels between employees assigned to mRNA‐ or Ad‐vector‐RVR. Some infections may not lead to seroconversion, which was not accounted for [21, 22].

mRNA‐RVR recipients consistently showed higher anti‐S IgG and neutralising capacity at 1 and 6 months post‐vaccine. According to FDA guidelines for convalescent plasma therapy, an anti‐spike IgG titre of ≥ 1.280 AU/mL is considered protective [23, 24]. However, Meschi et al. suggested a titre of 14,084.51 AU/mL as a robust vaccine‐induced immune response [25, 26]. Noteworthy, in our cohort, 12 months post‐RVR (i.e., 6 months post‐booster), only 57.1% and 29.5% of the mRNA and Ad‐vector vaccinated employees, respectively, exceeded this threshold of 14,084.51 AU/mL. Interestingly, these proportions were consistent across both Mann–Whitney U and regression analyses, despite some discrepancies in directionality elsewhere. As of now, there is no definitive cut‐off value established for anti‐spike IgG titre that reliably signifies robust protection against SARS‐CoV‐2 infection, a critical consideration in clinical settings, urging further investigation in research endeavours.

Although post‐vaccination, we observed a similar anti‐S IgG decrease over time among COVID‐19 naive and previously exposed individuals, the previously exposed individuals exhibited higher anti‐S titres 1 month after RVR completion, suggesting that previous infection results in a more enduring immune response post‐vaccination. A distinct pattern emerged, namely, neutralising antibodies in naive participants yet stable titres in those previously infected. This might be due to exposure to SARS‐CoV‐2 in different forms, the live virus and vaccine‐associated viral antigens. Nevertheless, despite a decay in anti‐S IgG, antibody functionality post‐infection remains remarkably high in both vaccination cohorts [27]. This is in contrast with numerous studies affirming a strong association between anti‐S antibodies and neutralising capacity [28, 29]. Our findings suggest this relationship may differ based on infection status before vaccination, as we conclude from the results in the COVID‐19 previously exposed group. Regression modelling confirmed the influence of prior exposure but did not always show significant differences in neutralising capacity at later visits. This suggests that infection history exerts a stronger influence on antibody levels than vaccine platform once baseline differences are controlled for.

Following booster vaccination, individuals with Ad‐vector‐RVR displayed lower anti‐S titres but higher neutralising capacity than individuals with mRNA‐RVR. Several articles suggest a more robust immune response following heterologous over homologous vaccination [30, 31]. While research often indicates comparable vaccine effectiveness between double mRNA vaccination and Ad‐vector/mRNA vaccination, it does not explain the persistently lower anti‐S levels in Ad‐vector vaccinated employees despite their significantly higher neutralising capacity. One possible explanation lies in the induction of better B‐cell maturation following the Ad‐vector vaccine, through early CD4+ T‐cell and broader B‐cell response [32]. Studies suggest that booster vaccination triggers affinity maturation, enhancing neutralising capacity against virus variants [33]. Assuming this effect is more pronounced after heterologous vaccination and considering the potentially superior maturation after Ad‐vector vaccination, we can account for the higher neutralising capacity observed in the Ad‐vector‐RVR participants post‐booster. Notably, this interpretation must be nuanced by the regression findings, which did not confirm significant differences in neutralising capacity post‐booster and, in some cases, indicated the opposite direction of effect for anti‐spike IgG compared to the Mann–Whitney U test. This underscores the importance of considering both analytical approaches when interpreting vaccine‐induced immune profiles. This heterologous effect was also observed within the mRNA‐RVR participants receiving a Spikevax booster. Despite these differences, anti‐NCP levels were comparable.

The size and duration of our follow‐up, together with the combination of different humoral immune assays, represent important strengths of this study.

Due to the limited availability of PCR testing at the study's initiation in May 2020, prior infection classification relied on serial anti‐NCP antibody measurements taken at 1, 6 and 9 months. As anti‐NCP IgG remains detectable for ±4.5 months after SARS‐CoV‐2 infection (data not shown), we were able to identify participants with relatively recent SARS‐CoV‐2 infection. The latter is of valuable importance because SARS‐CoV‐2 infection before vaccination influences the immune response post‐vaccination.

One of the limitations of this study is that, because of logistical constraints, the pre‐vaccination sampling was organised at the initiation of the mRNA vaccination campaign. Consequently, some individuals with Ad‐vector‐RVR might have contracted SARS‐CoV‐2 infection during the 6‐week interval between the pre‐vaccination sampling and the first Ad‐vector vaccine administration. Additionally, the difference in timing between the first and second doses (4 weeks for mRNA vs. 12 weeks for Ad‐vector) could have contributed to variations in immune response at Visit 5. Longer intervals between doses have been shown to enhance antibody affinity maturation and may partly explain the higher neutralising capacity observed post‐booster in the Ad‐vector group [34]. A second limitation is that our study cohort primarily comprises a healthy population, albeit with a notable sex imbalance, although typical within the healthcare context. Of note, the neutralising capacity of antibodies can vary depending on the encountered viral strain. As the assay we used is based on the Wuhan strain, vaccination‐induced antibodies were not assessed for neutralising potential against emerging VOCs (Figure 1). Consequently, a high neutralising capacity against the Wuhan strain may not necessarily correlate with efficacy against other variants, given their mutations in the RBD protein [35]. Moreover, this test specifically focuses on neutralising antibodies binding to the RBD, excluding antibodies that neutralise the virus by binding to other regions of the S‐protein [36].

Overall, our study provides insights into the dynamics of the humoral response against SARS‐CoV‐2 while highlighting that observed differences depend on both the primary vaccine platform and the statistical approach used. These findings underscore the need for a nuanced interpretation of booster responses, particularly in individuals with prior viral exposure. While our findings inform ongoing efforts to optimise vaccination strategies, the lack of a universally defined cut‐off for anti‐spike IgG limits the ability to determine precise protection thresholds. Future research should aim to establish such benchmarks and consider both unadjusted group‐level differences and covariate‐adjusted trends, in order to better guide vaccination and booster policies and to clarify the antibody levels required for robust protection against SARS‐CoV‐2.

## Author Contributions

**Els Van Nedervelde:** investigation, writing – original draft, methodology, validation, visualization, writing – review and editing, software, formal analysis, project administration, data curation. **Ellen Vancutsem:** conceptualization, investigation, funding acquisition, methodology, validation, writing – review and editing, project administration, supervision, resources. **Deborah De Geyter:** conceptualization, funding acquisition, methodology, investigation, writing – review and editing, project administration, supervision, resources. **Diederik De Cock:** writing – review and editing, data curation, formal analysis, visualization. **Rhea Buttiens:** conceptualization, investigation, data curation. **Thessa Laeremans:** writing – review and editing. **Joeri L. Aerts:** writing – review and editing, resources, supervision. **Sabine D. Allard:** conceptualization, funding acquisition, methodology, validation, visualization, writing – review and editing, project administration, supervision, resources.

## Funding

This study was supported by the UZ Brussel Foundation and Willy Gepts Fonds (Vrije Universiteit Brussel), SRP86 and the Pfizer Global Medical Grant (ID: 61646809).

## Conflicts of Interest

The authors declare no conflicts of interest.

## Supporting information


**Figure S1:** Comparison of participants' ages between different groups.
**Table S1:** Number of included employees per study visit and group.
**Table S2:** COVID‐19 naive/previously exposed distribution and anti‐NCP positive employees per booster subgroup.
**Table S3:** Q‐values from Mann–Whitney U analyses corresponding to the comparisons shown in Figures 2 and 3. The table presents comparisons between mRNA‐ and adenovector‐based vaccines across three participant groups: all participants, COVID‐19–naive participants and previously exposed participants. Analyses include anti‐spike IgG, anti‐nucleocapsid (NCP) IgG and neutralising antibody levels. Statistical significance was determined at a false discovery rate (FDR)–adjusted *q*‐value threshold of 0.05.
**Table S4:** Q‐values from Mann–Whitney U analyses corresponding to the comparisons shown in Figure 4. The table includes comparisons of anti‐spike IgG, anti‐nucleocapsid (NCP) IgG and neutralising capacity between mRNA‐ and Ad‐vector‐based vaccine recipients. It also includes comparisons between Pfizer and Moderna as booster vaccines, both overall and stratified by primary vaccination type (mRNA or Ad‐vector). Statistical significance was determined at a false discovery rate (FDR)–adjusted *q*‐value threshold of 0.05.
**Table S5:** irv_70202‐sup‐0001‐Appendix.docx. *p*‐values from multiple linear regression analyses corresponding to the comparisons shown in Figure 2 and 3. The table includes comparisons of anti‐spike IgG, anti‐nucleocapsid (NCP) IgG and neutralising capacity between mRNA‐ and Ad‐vector‐based vaccine recipients. It also includes comparisons after mRNA booster vaccines. The regression models included vaccine type as the main independent variable and adjusted for age and sex, with *p*‐values for these covariates also reported. Statistical significance was determined at a threshold of *p* < 0.05.
**Table S6:** irv_70202‐sup‐0001‐Appendix.docx. *p*‐values from multiple linear regression analyses (adjusted for age and sex) corresponding to the comparisons shown in Figure 4 after booster vaccination. The table includes comparisons of anti‐spike IgG, anti‐nucleocapsid (NCP) IgG and neutralising capacity between mRNA‐ and Ad‐vector‐based vaccine recipients, as well as between Pfizer and Moderna boosters, both overall and stratified by primary vaccination type (mRNA or adenovector). *p*‐values for the age and sex covariates included in the models are also reported. Statistical significance was determined at a threshold of *p* < 0.05.
**Table S7:** Unstandardised β coefficients with corresponding 95% confidence intervals (lower and upper limits) from multiple linear regression analyses (adjusted for age and sex) corresponding to the results in Appendix Table 5. Values are shown for anti‐spike IgG, anti‐nucleocapsid (NCP) IgG and neutralising capacity, comparing mRNA‐ and adenovector‐based vaccine recipients.
**Table S8:** Unstandardised β coefficients with corresponding 95% confidence intervals (lower and upper limits) from multiple linear regression analyses (adjusted for age and sex) corresponding to the booster vaccine subgroup comparisons in Appendix Table 5. Values are shown for anti‐spike IgG, anti‐nucleocapsid (NCP) IgG and neutralising capacity, comparing Pfizer and Moderna boosters, both overall and stratified by primary vaccination type (mRNA or adenovector).

## Data Availability

The data that support the findings of this study are available from the corresponding author upon reasonable request.
