# Supplementary material for: Longitudinal SARS‐CoV‐2 Antibody Response in Healthcare Workers: Benefit of Prior Infection and Heterologous Boosting on Anti‐Spike IgG Immunity
Source: Influenza Other Respir Viruses. 2026 Jan 5;20(1):e70202. doi: 10.1111/irv.70202 (PMC12771585; doi:10.1111/irv.70202)
Supplement: Supplementary file 1 — Figure S1: Comparison of participants' ages between different groups. Table S1: Number of included employees per study visit and group. Table S2: COVID‐19 naive/previously exposed distribution and anti‐NCP positive employees per booster subgroup. Table S3: Q‐values from Mann–Whitney U analyses corresponding to the comparisons shown in Figures 2 and 3. The table presents comparisons between mRNA‐ and adenovector‐based vaccines across three participant groups: all participants, COVID‐19–naive participants and previously exposed participants. Analyses include anti‐spike IgG, anti‐nucleocapsid (NCP) IgG and neutralising antibody levels. Statistical significance was determined at a false discovery rate (FDR)–adjusted q‐value threshold of 0.05. Table S4: Q‐values from Mann–Whitney U analyses corresponding to the comparisons shown in Figure 4. The table includes comparisons of anti‐spike IgG, anti‐nucleocapsid (NCP) IgG and neutralising capacity between mRNA‐ and Ad‐vector‐based vaccine recipients. It also includes comparisons between Pfizer and Moderna as booster vaccines, both overall and stratified by primary vaccination type (mRNA or Ad‐vector). Statistical significance was determined at a false discovery rate (FDR)–adjusted q‐value threshold of 0.05. Table S5: irv_70202‐sup‐0001‐Appendix.docx. p‐values from multiple linear regression analyses corresponding to the comparisons shown in Figure 2 and 3. The table includes comparisons of anti‐spike IgG, anti‐nucleocapsid (NCP) IgG and neutralising capacity between mRNA‐ and Ad‐vector‐based vaccine recipients. It also includes comparisons after mRNA booster vaccines. The regression models included vaccine type as the main independent variable and adjusted for age and sex, with p‐values for these covariates also reported. Statistical significance was determined at a threshold of p < 0.05. Table S6: irv_70202‐sup‐0001‐Appendix.docx. p‐values from multiple linear regression analyses (adjusted for age and sex) corresponding to [file IRV-20-e70202-s001.docx]

**APPENDIX**

**Appendix Figure 1:**


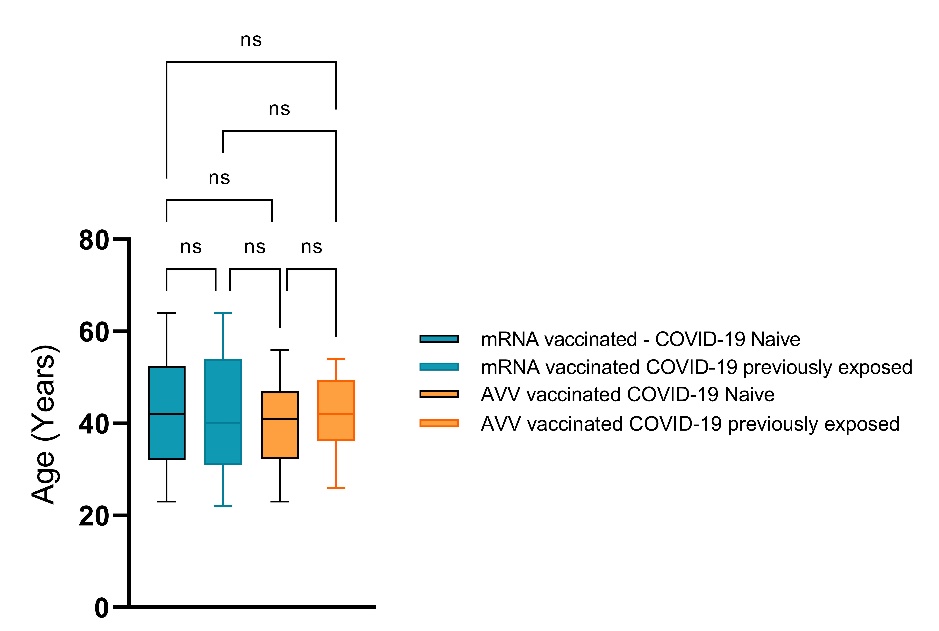


**Appendix Figure 1: Comparison of participants’ ages between different groups**

Age of employees receiving mRNA-RVR versus Ad-vector-RVR are compared. Participants are categorized into two groups: COVID-19 naive and COVID-19 previously exposed. Kruskal-Wallis test with significance denoted by a p-value below 0.05.

**Appendix Table 1:**

|  |  | **mRNA vaccinated employees** |  |  | **Ad-vector vaccinated employees** |  |  |
| --- | --- | --- | --- | --- | --- | --- | --- |
| **Visit** | **Total** | **COVID-19 naive** | **COVID-19 previously exposed** | **Total** | **COVID-19 naive** | **COVID-19 previously exposed** | **Total** |
| 4 | **380** | 289 | 91 | **200** | 164 | 36 | 580 |
| 5 | **380** | 289 | 91 | **168** | 138 | 30 | 548 |
| 6 | **304** | 228 | 76 | **162** | 130 | 32 | 466 |
| 7 | **300** | 229 | 71 | **150** | 119 | 31 | 450 |

**Appendix Table 1: Number of included employees per study visit and group**

*This table represents the number of participating employees per study visit. The data is organized based on vaccination groups, with subdivisions for COVID-19 naive and COVID-19 previously exposed employees within each group. Ad-vector, Adenoviral vector vaccine.*

**Appendix Table 2:**

|  |  | **Total** |  |  | **mRNA vaccinated** |  |  | **Ad-vector vaccinated** |  |
| --- | --- | --- | --- | --- | --- | --- | --- | --- | --- |
|  | **Spikevax®** | **Comirnaty®** | **No booster** | **Spikevax®** | **Comirnaty®** | **No booster** | **Spikevax®** | **Comirnaty®** | **No booster** |
| **Natural infection before RVR** | | | | | | | | | |
| **COVID-19 naive** | **311** | 34 | 3 | **197** | 29 | 3 | 114 | 5 | **0** |
| **COVID-19 previously exposed** | **89** | 12 | 1 | **61** | 10 | 0 | 28 | 2 | **1** |
| **Anti-NCP positive after RVR** | | | | | | | | | |
| **Visit 5** | **33** | 6 | 0 | **23** | 5 | 0 | 10 | 1 | **0** |
| **Visit 6** | **24** | 1 | 0 | **15** | 1 | 0 | 9 | 0 | **0** |
| **Visit 7** | **89** | 8 | 2 | **47** | 6 | 1 | 42 | 2 | **1** |

**Appendix Table 2: COVID-19 naive/previously exposed distribution and anti-NCP positive employees per booster subgroup**

*Upper part: distribution of COVID-19 naive and COVID-19 previously exposed employees within each booster group, both in total and per basic vaccination regimen.*

*Lower part: number of employees positive for anti-NCP IgG antibodies, indicative of recent natural infection, per visit and subgroup.*

Appendix table 3:

|  |  | **q-values** |  |
| --- | --- | --- | --- |
|  | **Anti-spike IgG (AU/ml)** | **Neutralising antibodies (%)** | **Anti-NCP IgG (S/Co)** |
| **All participants** | | | |
| **Visit 4** | 0,053301 | **0,040147** | 0,425508 |
| **Visit 5** | **<0,000001** | **<0,000001** | 0,425508 |
| **Visit 6** | **<0,000001** | 0,000448 | 0,935466 |
| **Visit 7** | **<0,000001** | **<0,000001** | 0,384016 |
| **COVID-19 naive participants** | | | |
| **Visit 4** |  |  | 0,950734 |
| **Visit 5** | **<0,000001** | **<0,000001** | **0,02257** |
| **Visit 6** | **<0,000001** | **0,003433** | 0,259055 |
| **Visit 7** | **<0,000001** | **<0,000001** | 0,259055 |
| **COVID-19 previously exposed** | | | |
| **Visit 4** | 0,159902 | 0,214117 | 0,517746 |
| **Visit 5** | **<0,000001** | 0,714952 | 0,517746 |
| **Visit 6** | **0,022354** | **0,011945** | 0,517746 |
| **Visit 7** | 0,107477 | **0,011945** | 0,517746 |

**Appendix Table 3:** Q-values from Mann–Whitney U analyses corresponding to the comparisons shown in Figures 2 and 3. The table presents comparisons between mRNA- and adenovector-based vaccines across three participant groups: all participants, COVID-19–naive participants, and previously exposed participants. Analyses include anti-spike IgG, anti-nucleocapsid (NCP) IgG, and neutralising antibody levels. Statistical significance was determined at a false discovery rate (FDR)–adjusted q-value threshold of 0.05.

Appendix table 4:

|  |  | **q-values** |  |
| --- | --- | --- | --- |
|  | **Anti-spike IgG (AU/ml)** | **Neutralising antibodies (%)** | **Anti-NCP IgG (S/Co)** |
| **Comparison based on recommended vaccination schedule** | | | |
| **Visit 7** | **<0,000001** | **<0,000001** | 0,10879 |
| **Comparison based on booster type** | | | |
| **Visit 7** | **0,017658** | 0,099842 | 0,589039 |
| **Comparison based on booster type within mRNA vaccinated employees** | | | |
| **Visit 7** | **0,000018** | 0,388675 | 0,635771 |
| **Comparison based on booster type within Ad-vector vaccinated employees** | | | |
| **Visit 7** | 0,648958 | 0,710051 | 0,897089 |

**Appendix Table 4:** Q-values from Mann–Whitney U analyses corresponding to the comparisons shown in Figure 4. The table includes comparisons of anti-spike IgG, anti-nucleocapsid (NCP) IgG, and neutralising capacity between mRNA- and Ad-vector-based vaccine recipients. It also includes comparisons between Pfizer and Moderna as booster vaccines, both overall and stratified by primary vaccination type (mRNA or Ad-vector). Statistical significance was determined at a false discovery rate (FDR)–adjusted q-value threshold of 0.05.

Appendix table 5:

|  | **p-value** | | | | | | | | |
| --- | --- | --- | --- | --- | --- | --- | --- | --- | --- |
|  | **Anti-spike IgG** | | | **Neutralising antibodies** | | | **Anti-NCP IgG** | | |
|  | **(AU/ml)** | | | **(%)** | | | **(S/Co)** | | |
|  | **mRNA vs Ad-Vector** | **Sex** | **Age** | **mRNA vs Ad-Vector** | **Sex** | **Age** | **mRNA vs Ad-Vector** | **Sex** | **Age** |
| **All participants** | | | | | | | | | |
| **Visit 4** | 0,313 | 0,059 | 0,121 | 0,224 | 0,094 | 0,141 | 0,496 | 0,55 | 0,45 |
| **Visit 5** | **<0,001** | 0,143 | **0,018** | **<0,001** | 0,493 | 0,064 | 0,438 | 0,096 | 0,43 |
| **Visit 6** | **0,009** | 0,4 | **0,04** | **0,007** | 0,849 | **0,005** | 0,608 | 0,695 | 0,918 |
| **Visit 7** | **<0,001** | 0,607 | 0,982 | 0,104 | 0,712 | 0,924 | 0,08 | 0,112 | 0,177 |
| **COVID-19 naive participants** | | | | | | | | | |
| **Visit 4** |  |  |  |  |  |  | 0,492 | 0,562 | 0,998 |
| **Visit 5** | **<0,001** | 0,74 | **<0,001** | **<0,001** | 0,89 | 0,124 | **<0,001** | 0,406 | 0,712 |
| **Visit 6** | 0,127 | 0,804 | 0,67 | **0,018** | 0,147 | **0,001** | **0,044** | 0,797 | 0,411 |
| **Visit 7** | 0,147 | 0,896 | 0,866 | **<0,001** | 0,718 | 0,769 | 0,184 | 0,093 | 0,054 |
| **COVID-19 previously exposed** | | | | | | | | | |
| **Visit 4** | 0,449 | **0,029** | 0,093 | 0,144 | **0,037** | 0,084 | 0,524 | 0,18 | 0,408 |
| **Visit 5** | **<0,001** | 0,082 | 0,136 | 0,769 | 0,517 | 0,227 | 0,597 | 0,253 | 0,104 |
| **Visit 6** | **0,041** | 0,487 | **0,003** | 0,747 | 0,395 | 0,824 | 0,323 | 0,679 | 0,152 |
| **Visit 7** | 0,099 | 0,443 | 0,593 | 0,443 | **0,018** | 0,31 | 0,183 | 0,912 | 0,935 |

**Appendix Table 5:** p-values from multiple linear regression analyses corresponding to the comparisons shown in Figure 2 and 3. The table includes comparisons of anti-spike IgG, anti-nucleocapsid (NCP) IgG, and neutralising capacity between mRNA- and Ad-vector-based vaccine recipients. It also includes comparisons after mRNA booster vaccines. The regression models included vaccine type as the main independent variable and adjusted for age and sex, with p-values for these covariates also reported. Statistical significance was determined at a threshold of p < 0.05.

|  | **p-value** | | | | | | | | |
| --- | --- | --- | --- | --- | --- | --- | --- | --- | --- |
|  | **Anti-spike IgG** | | | **Neutralising antibodies** | | | **Anti-NCP IgG** | | |
|  | **(AU/ml)** | | | **(%)** | | | **(S/Co)** | | |
|  | **Booster brand** | **Sex** | **Age** | **Booster brand** | **Sex** | **Age** | **Booster brand** | **Sex** | **Age** |
| **Comparison based on recommended vaccination schedule** | | | | | | | | | |
| **Visit 7** | 0,099 | 0,443 | 0,593 | 0,443 | **0,018** | 0,31 | 0,183 | 0,912 | 0,935 |
| **Comparison based on booster type** | | | | | | | | | |
| **Visit 7** | **0,009** | 0,763 | 0,565 | 0,633 | 0,787 | 0,772 | 0,892 | 0,081 | 0,227 |
| **Comparison based on booster type within mRNA vaccinated employees** | | | | | | | | | |
| **Visit 7** | **<0,001** | 0,93 | 0,743 | 0,864 | 0,77 | 0,976 | 0,838 | 0,184 | 0,517 |
| **Comparison based on booster type within Ad-vector vaccinated employees** | | | | | | | | | |
| **Visit 7** | 0,386 | 0,821 | 0,577 | 0,082 | **0,016** | 0,71 | 0,738 | 0,211 | 0,293 |

***Appendix Table 6:*** *p-values from multiple linear regression analyses (adjusted for age and sex) corresponding to the comparisons shown in Figure 4 after booster vaccination. The table includes comparisons of anti-spike IgG, anti-nucleocapsid (NCP) IgG, and neutralising capacity between mRNA- and Ad-vector-based vaccine recipients, as well as between Pfizer and Moderna boosters, both overall and stratified by primary vaccination type (mRNA or adenovector). p-values for the age and sex covariates included in the models are also reported. Statistical significance was determined at a threshold of p < 0.05.*

|  |  | | | | | | | | |
| --- | --- | --- | --- | --- | --- | --- | --- | --- | --- |
|  | **Anti-spike IgG** | | | **Neutralising antibodies** | | | **Anti-NCP IgG** | | |
|  | **(AU/ml)** | | | **(%)** | | | **(S/Co)** | | |
|  | **Unstanderdised Beta** | **Upper limit** | **Lower limit** | **Unstanderdised Beta** | **Upper limit** | **Lower limit** | **Unstanderdised Beta** | **Upper limit** | **Lower limit** |
| **All participants** | | | | | | | | | |
| **Visit 4** | 46,264 | -43,551 | 136,079 | 1,277 | -0,785 | 3,339 | -0,83 | -0,322 | 0,156 |
| **Visit 5** | -10566,1 | -12076 | -9056,18 | -6,905 | -8,759 | -5,05 | 0,07 | -0,107 | 0,247 |
| **Visit 6** | -944,746 | -1653,53 | -235,963 | -4,011 | -6,91 | -1,113 | 0,042 | -0,119 | 0,204 |
| **Visit 7** | -7153,4 | -9098,95 | -5207,85 | 1,197 | -0,246 | 2,64 | 0,328 | -0,04 | 0,697 |
| **COVID-19 naive participants** | | | | | | | | | |
| **Visit 4** |  |  |  |  |  |  | -0,009 | -0,034 | 0,016 |
| **Visit 5** | -8577,57 | -9784,23 | -7370,91 | -8,237 | -10,31 | -6,165 | 0,21 | 0,103 | 0,317 |
| **Visit 6** | -455,888 | -1040,86 | 129,086 | -3,798 | -6,947 | -0,649 | 0,156 | 0,004 | 0,308 |
| **Visit 7** | -8287,43 | -10505,3 | -6069,6 | 1,309 | -0,459 | 3,077 | 0,226 | -0,107 | 0,559 |
| **COVID-19 previously exposed** | | | | | | | | | |
| **Visit 4** | 165,288 | -262,498 | 593,075 | 6,291 | -2,144 | 14,726 | 0,265 | -0,551 | 1,082 |
| **Visit 5** | -16026,4 | -19807,8 | 12244,95 | -0,563 | -4,327 | 3,201 | -0,177 | -0,835 | 0,481 |
| **Visit 6** | -2206,49 | -4323,85 | -89,126 | -0,532 | -3,76 | 2,696 | -0,228 | -0,68 | 0,224 |
| **Visit 7** | -3321,46 | -7270,37 | 627,448 | 0,61 | -0,949 | 2,0169 | 0,756 | -0,356 | 1,868 |

***Appendix Table 7:*** *Unstandardised β coefficients with corresponding 95% confidence intervals (lower and upper limits) from multiple linear regression analyses (adjusted for age and sex) corresponding to the results in Appendix Table 5. Values are shown for anti-spike IgG, anti-nucleocapsid (NCP) IgG, and neutralising capacity, comparing mRNA- and adenovector-based vaccine recipients.*

|  | **p-value** | | | | | | | | |
| --- | --- | --- | --- | --- | --- | --- | --- | --- | --- |
|  | **Anti-spike IgG** | | | **Neutralising antibodies** | | | **Anti-NCP IgG** | | |
|  | **(AU/ml)** | | | **(%)** | | | **(S/Co)** | | |
|  | **Unstanderdised Beta** | **Upper limit** | **Lower limit** | **Unstanderdised Beta** | **Upper limit** | **Lower limit** | **Unstanderdised Beta** | **Upper limit** | **Lower limit** |
| **Comparison based on recommended vaccination schedule** | | | | | | | | | |
| **Visit 7** | -7153,4 | -9098,95 | -5207,85 | 1,197 | -0,246 | 2,64 | 0,328 | -0,04 | 0,697 |
| **Comparison based on booster type** | | | | | | | | | |
| **Visit 7** | 5471,102 | 1358,9 | 9583,305 | 0,701 | -2,179 | 3,581 | 0,046 | -0,623 | 0,715 |
| **Comparison based on booster type within mRNA vaccinated employees** | | | | | | | | | |
| **Visit 7** | 9523,782 | 5396,036 | 13651,53 | 0,057 | -3,704 | 3,818 | -0,074 | -0,782 | 0,634 |
| **Comparison based on booster type within Ad-vector vaccinated employees** | | | | | | | | | |
| **Visit 7** | -5027,74 | -16392,8 | 6337,363 | 2,214 | -0,277 | 4,706 | 0,349 | -1,696 | 2,395 |

***Appendix Table 8:*** *Unstandardised β coefficients with corresponding 95% confidence intervals (lower and upper limits) from multiple linear regression analyses (adjusted for age and sex) corresponding to the booster vaccine subgroup comparisons in Appendix Table 5. Values are shown for anti-spike IgG, anti-nucleocapsid (NCP) IgG, and neutralising capacity, comparing Pfizer and Moderna boosters, both overall and stratified by primary vaccination type (mRNA or adenovector).*
